# Maximising inclusivity in care home research: Lessons learned from the AFRI-c randomised controlled trial

**DOI:** 10.1016/j.tjfa.2025.100038

**Published:** 2025-04-05

**Authors:** Laurel Campbell-Smith, Sophie Rees, Jane Sprackman, Karen Sargent, Alastair D Hay, Rachel CM Brierley

**Affiliations:** a Bristol Trials Centre, Bristol Medical School, University of Bristol, Bristol, UK; b Patient and public contributor, UK; c Centre of Academic Primary Care, Bristol Medical School, University of Bristol, Bristol, UK

**Keywords:** Consent, Capacity, Care home, Barriers, Consultee

## Abstract

Ethical and procedural requirements make research in care homes challenging. With people living longer globally, it is essential that older people are included in research, including within the care home setting. We conducted a randomised controlled trial (AFRI-c) in 91 care homes across England, aiming to make the study available to every eligible resident. Facilitators included flexible models for receiving consent; commitment from care home staff, residents and families; tailored and specific training for care home staff; and support from national research infrastructure to engage care homes in research. To facilitate inclusive care home research, we recommend consulting with care homes about their research priorities; continuing investment in national research infrastructure for care homes; using advance directives for research planning for care home residents; embedding research nurses in care home environments; and more guidance for researchers and ethics committees on applying legal frameworks regarding capacity to research settings.

## 1 Introduction

The challenges of conducting research in care homes have historically led to the exclusion of the setting and the residents and staff living and working within them [1]. However, with an ageing population within the UK and globally [2], their absence from research is increasingly seen as problematic. The COVID-19 pandemic further highlighted the vulnerability of this under-researched population [3]. Research has previously been hampered by a lack of robust research infrastructure in care homes [4], and disrupted their everyday activities and support networks [[1], [2], [3]]. Even where research is undertaken in care homes, many researchers choose to exclude residents who do not have capacity to consent for themselves [4].

Research has previously been hampered by a lack of robust research infrastructure in care homes [5]. In the UK, the National Institute for Health and Care Research (NIHR), funded by the Department of Health and Social Care, recently increased its focus on social care research and has been investing to establish infrastructure to address this [6]. One key obstacle to conducting research in care homes is the consent process, particularly for residents who lack the capacity to consent to research [7,8]. Residents without capacity to consent are seldom included in care home research because the ethical, moral and procedural challenges of involving them are often seen as too great [9]. The result is that an important and large demographic is omitted from research which could benefit them [10].

The ‘Air Filtration to prevent symptomatic winter Respiratory Infections (including COVID-19) in care homes (AFRI-c)’ study was designed to encourage the participation of residents, regardless of capacity. It was as inclusive as possible because people living with dementia might benefit more from preventing infections (for example through fewer delirious episodes caused by infection) [11]. Implementing this inclusive design was challenging. From our experiences of delivering this large cluster randomised controlled trial (RCT) in care homes, we identify key challenges around consenting older residents for research and make recommendations to support future research.

## 2 Study design

The study design has already been reported elsewhere in detail [12]. The study aimed to determine whether use of high-efficiency-particulate-air (HEPA) filtration units could reduce symptomatic respiratory infections including COVID-19 in care homes. It was designed during the COVID-19 pandemic to allow remote recruitment, intervention delivery and data collection. Over three consecutive winters, 91 care homes in England were randomised to receive HEPA filtration units for bedrooms and communal areas (intervention) or to continue with normal infection control measures (control). At all care homes, 10–16 residents (selected at random) were invited to give consent to allow access to their medical records for the study period. Additionally, these 10–16 residents in the intervention group were invited to consent to having a HEPA filtration unit in their private bedroom for one winter period (up to 9 months). Additional residents were consented to replace residents leaving the study for any reason, to maintain a steady number of consented residents at each care home. Ethical approval was given by the London - Harrow Research Ethics Committee (REC, Ref: 21/HRA/4318) for the study.

The Enabling Research in Care Homes (ENRICH) and Research Delivery Network (RDN) are funded by the NIHR. These teams facilitate research, including in care homes, by employing teams of research nurses and research practitioners to deliver clinical research across England. Similar organisations exist in UK devolved nations. This support was crucial to the study's success.

In the following sections we explore the challenges we experienced around consenting older residents in care homes, drawing on reflections of the study team.

### 2.1 Flexible consent models

As the study was set-up during the COVID-19 pandemic, we needed to facilitate obtaining consent when care homes were experiencing lockdowns and visitors were not allowed access to the home. The AFRI-c team developed multiple methods for obtaining informed consent, including face-to-face consultations using paper consent forms (preferred method for residents with capacity); e-Consent forms; and verbal consent. The consent methods and their benefits/challenges are summarised in Table 1. Flexibility was essential to inclusive recruitment. Delegated care home staff were permitted to receive informed consent. Depending on care home preferences, the study research nurse / clinical study officer, trained care home staff, research delivery teams (or a combination) received informed consent / advice from consultees. Some care homes opted not to consent due to lack of staff time, but others appreciated it as an opportunity to participate in continuing professional development.

**Table 1:** Benefits and challenges of the different consent methods used in AFRI-C.

Method of Consent	Process	Benefits	Challenges
Resident E-Consent	• Resident provided with study information (usually by care home staff) • Resident approached by research/care home staff • Informed consent conversation with resident completed • E-consent form presented to resident on laptop/study iPad • Resident asked to click each statement to indicate consent • Copy of completed E-consent form provided to resident, Care Home and GP practice	• Removed the requirement for “Wet signature” reducing visits needed to homes (important during lockdown) • E-Consent forms were user friendly and designed to reduce risk of incorrect completion	• Copies of E-consent forms were often not stored in the care home site file and care homes record as required • Oversight and training were required to ensure that care home staff were not completing E-consent forms on behalf of residents
Consultee* E-Consent	• Care home obtained permission from Consultee to share their contact details with research team • Consultee contacted by research team via text/email/call • Consultee information pack sent via email • Link to E-consent form sent via email just before the call and E-consent form completed together with researcher during phone call/video call • Copy of completed E-consent form provided to Consultee, care home and GP	• Reduced risk of original consent forms being handled inappropriately/lost • Most care homes used electronic records systems. E-forms were easier to add to residents’ care notes • Alternative method for residents/Consultees who struggled with holding a pen or had eyesight problems • Removed need to scan paper copies to upload to the study database or send to GP practices • Facilitated consent form checks in near “real time” • Enabled Consultees not local to care home to undertake the Consultee role (**C**) • Removed reliance on slow and potentially unreliable postal systems where Consultees were not local to the care home (**C**)	• Required email address, access to computer device and ability to navigate Microsoft Forms. Common for older spouses to not meet these criteria (**C**) • E-forms were sometimes completed before the Consent conversation had taken place, resulting in Consultees having to be contacted multiple times (**C**) • Consultees were required to enter the residents ID numbers into the E-consent forms which led to errors (**C**)
Face to Face consent	• Information sheets passed to selected residents/Consultee, usually by care home staff • Trained care home/RDN assisted residents/Consultees were permitted to receive informed consent • If resident/Consultee wished to participate, paper consent form completed with wet signatures and informed consent was received • Copies were provided to resident/Consultee, care home notes and GP; original stored in site file	• Paper consent forms are used as standard within research • Having face to face assessments is regarded as best practise within research • RDN staff able to demonstrate in person how to receive informed consent to care home staff • Ability to intervene if concerns with how care homes were completing the consenting	• Consent forms were not reliably completed in line with GCP: - Care home staff initialled/ signed on behalf of residents/Consultees - Incorrect information sheet date and version number recorded - Incorrect versions of consent forms used - Amendments made without date and initials/with Tippex - Consent forms not treated like legal documents • Original consent forms lost, shredded and not stored in site file • Time consuming to manually check paper consent forms across many sites
Remote paper consent	• 2 x copies of the Consultee declaration form were posted (pre-signed by researcher) Consent conversation completed over a phone/video call Consultee signed both copies of consent form returned one to study team via post	• Alternative method for those not living locally who were unable to use e-forms • Flexible process for Consultees, which did not require them to commit to care homes visits or multiple calls • Contact free method, useful during lockdown	• Postal consent process lengthy/relied on post • Risk of lost original consent forms • Poor uptake of video calls which removed the face-to-face assessment of the consent process/conversation • Complications around who would pay for postage and how when completed by RDN teams. • Some research nurses were uncomfortable with pre-signing consent forms

*Consultee = Person with close personal or professional relationship to resident who provides advice on residents wishes regarding their participation in the research; (C) = Relevant to Consultee consents only.

Throughout the study, trained local RDN/ENRICH research delivery staff supported the consent process in-person and remotely. The available support varied geographically, meaning not all care homes received the same level of assistance with consent processes. Care homes without regular local support relied upon remote guidance from the central study team, which made communicating information and research processes more challenging and resource intensive.

To ensure care home staff received suitable study training, the central study team created short online training videos split into modules. Modules were completed according to tasks assigned on the delegation log. Training included how to assess capacity for consent and incorporated required elements of Good Clinical Practice (GCP) relevant to the delegated study tasks, e.g. receiving informed consent. This removed the need for care home staff to complete formal GCP training, which would have been burdensome on care home staff and could have resulted in poor uptake of care homes agreeing to be involved in the study.

Allowing care home staff to receive consent presented some challenges and highlighted some wider issues with conducting research in care homes, which we discuss in the following sections.

### 2.2 Assessing capacity to consent

The Mental Capacity Act 2005 (MCA) is a legal framework that applies in England and Wales to support and empower people who are unable to make some or all decisions for themselves [13]. The MCA is written to include these individuals in the decision-making process wherever possible and ensure that decisions and choices about their care are made in line with their past and present wishes, beliefs and values. This includes decisions about consenting to research. However, in the research context, the operationalisation of the MCA can be subjective and lead to uncertainty for researchers [14].

In AFRI-c, care home staff were asked to inform the research team if they had concerns about the resident's capacity to consent to the study. On occasion experienced research delivery staff disagreed with care home staff's feedback regarding their residents capacity to consent. These differing evaluations of individual residents’ capacity could be explained in several ways.

The MCA stipulates that a person's capacity is time and decision specific [13]. Staff may have been influenced by the ability of the resident to make simple day-to-day decisions within the home (e.g. about food) but not considered their capacity in regards to the specific question of research and their ability to weigh up the benefits and risks of taking part in this research and to retain the information. They may also have struggled with operationalisation of the MCA. For example, current guidelines stipulate a two-stage test when assessing capacity [13]. Stage two requires the person receiving consent to ascertain if the participant can ‘retain/weigh the information’ and ‘communicate this, by any means’ . Our experiences were that care home staff struggled with the assessment of a residents’ ability to retain information. However, there is no explicit time frame stated by the MCA regarding how long individuals should retain information to be consideredcapacitous [15]. Similarly, communication ‘by any means’ puts an onus on the person receiving informed consent to consider if the person assents or dissents to research. They must attempt to understand the potential participant's verbal or non-verbal cues, to make an assessment on their capacity to consent to research This may be difficult in the context of assessing capacity in those living with dementia or fluctuating capacity [16]. The differences in training and function between care home staff and other health care professionals [17] may explain why the subjective elements of the MCA led to discrepancies in assessment of capacity for consent throughout the study.

If, during site visits, the ENRICH/RDN delivery teams had concerns about the application of the MCA, these would be flagged to the central study team who provided advice and guidance. In such situations care homes were usually asked to pause consenting until a review could be undertaken. Delivery teams made the final assessment of the resident's capacity for consent. Differing capacity assessments led to delays in the consenting process and resulted in delivery teams having to make multiple visits to sites.

The application of the MCA for fluctuating capacity in care homes was also challenging. The MCA states ‘there is no need for the researcher to monitor capacity proactively’ and that capacity should be assumed unless established otherwise. It also states that ‘consent is generally not valid following loss of capacity’ and researchers need to seek advice from a consultee in the event of a loss of capacity. In the context of conducting research in care homes, where it is likely that participants may experience fluctuating (or loss of) capacity, this raises the question about who is responsible for monitoring capacity of participants and how this should be managed. This was especially relevant in a study like AFRI-c that did not include ongoing participant involvement, so there were limited opportunities to re-assess capacity unless specific concerns were raised by care home staff or research staff visiting the care home.

### 2.3 Gatekeeping

Care home staff can both facilitate and block access to research in care homes, commonly referred to as “gatekeeping” [18]. Gatekeepers can enable access to research (e.g. by signing up the care home to participate in relevant research). They can also impede research, e.g. some eligible residents were never given the chance to participate because of gatekeeping by care home staff. Where access to residents was denied, it was usually due to the care home staff protecting the residents from perceived harm. This highlighted the tension between preserving a resident's autonomy and protecting residents from harm [19]. We experienced a high level of gatekeeping across many care homes, which may have had a negative impact on the variety and inclusivity of our participant resident pool. Our experience supports previous research findings that some care home staff hold paternalistic attitudes towards the residents who live within a care home and their relatives [20].

There is a need to balance residents’ autonomy about taking part in research versus respecting the care home's assessment on the appropriateness to approach. Managing gatekeeping was particularly challenging where concerns had to be addressed via email or telephone in care homes where there was lack of in-person research support. On the other hand, the close (sometimes family-like) relationships between residents and care home staff [21] could have influenced residents' decision-making. Residents may have been reluctant to decline to participate through fear of not wanting to disappoint their caregivers [22]. These close relationships could also have made the capacity assessments and consent discussions challenging for some care home staff.

### 2.4 Use of consultees

For residents lacking capacity to consent, we sought advice on the resident's wishes about participation in the research from a suitable consultee (ideally a personal consultee). A personal consultee is a close family member or friend of the resident. A nominated consultee is someone with a professional relationship to the resident who was independent from the study, such as a GP or solicitor*.*

Initially, the Research Ethics Committee (REC) did not approve the use of nominated consultees (originally we proposed care home staff could act as nominated consultees). Due to this restriction, some residents who lacked capacity to consent and did not have a next of kin were denied the opportunity to participate in Winters 1 and 2. During the set-up of Winter 2 an entire care home was unable to participate because most residents lacked capacity to consent and had no suitable personal consultee. The link between socioeconomic class and close familial relationships is well documented [23] and the exclusion of residents with neither capacity nor personal consultee suggested that an important and under-served demographic of residents were being excluded from the study. The study team removed the proposal for care home staff to act as nominated consultees and the REC subsequently approved use of nominated consultees during Winter 2. However, this amendment was not as useful as the study team had hoped. Health professionals (e.g. General Practitioners) sometimes declined to fulfil the role of nominated consultee due to a perceived conflict of interest, despite having no known connection to the study. Solicitors also declined on the basis that they would not undertake the role without financial recompense.

The use of consultee declarations for participants who lack capacity can be considered problematic as they are not person-centred, and best practice for the consent process should involve some face-to-face discussion with all stakeholders included [24]. However, during the delivery of AFRI-c there was an immediate threat of COVID-19. This meant that a collaborative discussion with residents and consultees was not always possible, and communication between staff and consultees was often done remotely via telephone, text, or email. The following strategies were utilised to try and ensure that the resident's wishes were central to the consultee declaration process.

• Consultees were reminded that the question was not whether *they* themselves would want the resident to be participating in the study, but to consider the information and offer advice about whether participation would have been something the resident would likely have wanted, using their personal knowledge of the resident.
• Where consultees felt they could not make this assessment or if they felt the resident would have not wanted to participate, then the resident was not included.

On occasion, particularly if the consultee role was being fulfilled by a spouse, it was found that the consultee themselves did not have capacity, which meant an alternative consultee was sought.

Our experiences highlight how the legal and ethical requirements in research sometimes lead to restrictions that exclude the people they are designed to protect. Clinical trial participants are often not representative of those most in need, including older adults [25,26]. As research in the care home sector grows, work is needed to facilitate adaptive and inclusive consent processes that can be replicated in future social care research. In AFRI-c, 169 of 1243 (14 %) of residents without capacity to consent were reported as having no suitable personal or nominated consultee, so were unable to participate. However, 709 of 1169 (61 %) of all AFRI-c participants were included on the advice of consultees, which demonstrates the feasibility of including residents/participants without capacity for consent in large-scale RCTs.

### 2.5 Understanding research principles

All care home staff received tailored and specific training for AFRI-c. However, their understanding of basic principles of research and willingness to adhere to such principles varied. For example, mitigation of selection bias by randomly selecting residents to approach for the study was questioned by some care home staff. One care home initially disregarded the list of randomly selected residents entirely and consented residents they thought would be ‘easiest’ to consent.

During the study, there were frequent protocol deviations, especially in research naïve care homes. Protocol breaches mostly involved sharing identifiable information about residents via non-secure email. Other breaches included inappropriate storage of essential study documents, shredding original consent forms and staff completing consents which they had not been delegated to undertake. The busy nature of a care home environment meant some staff aimed to complete research tasks as quickly as possible. When errors were made (e.g. incorrectly completed consent forms), tasks would need to be redone, which was frustrating for care home staff who are already working in busy and high-pressured environments. Researchers using plain language to explain complex research processes was crucial to try to avoid these situations. Where care staff were very engaged with and supportive of the research, fewer issues occurred.

## 3 Discussion

As research in care homes increases, it is essential for researchers to share our approaches to tackling perceived barriers in care home research. We hope this paper will support other researchers to aim to include all residents within a care home setting, regardless of frailty, capacity and socioeconomic status, in a wide range of research. Whilst the AFRI-c study was designed to be deliverable during the COVID-19 pandemic, the consent methods adopted for the study are also applicable to current trials, e.g. those using decentralised models. The lessons learned from conducting an inclusive study in care homes are relevant to other care home studies taking place outside a pandemic. The ethical and procedural regulations around research are sometimes regarded as a barrier to progress within medical research [27] and have been critiqued by researchers as “tiresome and wasteful” [28]. The ethics of conducting research in the vulnerable care home population is complex, particularly when including residents who lack capacity to consent. The UK government launched a review in 2021 to reduce the level of ‘red tape’ in research [29]. In our experience, the steps required to ensure the safety and wellbeing of participants involves a complicated consent process that busy and research-naïve care home staff sometimes found hard to follow. The stringent requirements surrounding the completion of consent forms and managing variations of information sheets (e.g. for consultees and residents with capacity) are important aspects of research governance but are time-consuming and regarded as unnecessary by some care home staff. Providing more explanation and training on why these requirements exist may have been useful but providing rationale alone may not be enough to reduce perceptions of burdensome rules [30].

Another key challenge around consenting residents in care homes is the application of the MCA to correctly assess capacity for consent. The Health Research Authority (HRA) code of practice [31] provides additional guidance on how to apply the MCA in complex areas, however, further clarity on applying the MCA in a social care setting would strengthen the available research infrastructure and provide clarity on situations arising from conducting research in care homes, such as fluctuating capacity. It is accepted that advance care planning will play a key role when addressing the problems associated with dementia care in an aging population [32]. This could be expanded to include decisions about research. Advance research planning (ARP) has been proposed to improve the involvement of people lacking capacity in research. ARP is a documented preference regarding research which can provide residents with dementia and those who are likely to experience impaired capacity an opportunity to discuss their research preferences [33,34]. Gatekeeping was a considerable challenge during the delivery of AFRI-c, and some consultees were unsure about residents’ views or were unavailable to provide advice. An existing ARP may have reduced some of the burden, for staff, families and next of kin when asked about a resident's preferences about research. Several obstacles to implementing ARP in practice have been identified and further work is needed to develop interventions to facilitate communication and embed ARP [35], and to consider the right time to have such conversations. However, adding preferences about research into advance care planning could preserve residents’ autonomy over research decisions [33,34].

We found care homes were generally very receptive to being involved with research. Care homes were particularly keen on supporting research to fight back against COVID-19, following the challenges faced in the pandemic. We recommend that researchers actively engage with care homes about what they see as the key research priorities, and consider the individual context within each care home [36]. In addition, if research in care homes is to become more commonplace then care homes need to receive support to enable research principles to be embedded into care homes through a change of culture, as has been happening within the NHS [37]. This would make it easier for care homes to understand fundamental research concepts. For AFRI-c, study set-up and delivery were less challenging when the care home team had some research experience. It has been suggested that embedding trained research staff into healthcare settings can influence research participation within these settings at both a team and individual level [38]. Most NHS settings participating in research have the active support of local embedded research nurse/s. Whilst there is some support for care homes to conduct research through the NIHR RDN, including online training resources, our experience was that in-person support varied regionally depending on local resources and other research commitments. It is unrealistic to expect busy care home staff to have time to conduct research without additional staff. We recommend a combined solution is needed to address this complex challenge: (i) continuing to invest in national resource infrastructure to promote and support research in the care home sector, such as the ENRICH network; (ii) consulting with care homes about their research priorities; and (iii) embed research nurses in care home settings to promote a culture for research; (iv) researchers should simplify research processes and language, wherever possible, to make it easier for care homes to get involved in research.

### 3.1 Recommendations

• Ensure that studies for care home settings are designed with flexible methods for consent to enable varying care home settings to participate.
• Embed research nurses in care home environments to support a change in culture towards care homes participating in more research.
• Continue to invest in national research infrastructure for care homes, such as the ENRICH network.
• Consult with care homes about their research priorities.
• Introduce advance research planning as part of care homes’ initial resident assessment paperwork.
• Improved guidance for researchers and ethics committees on application of the MCA in research, including the use of both personal and nominated consultees.
• Simplify research processes and language.

### 3.2 Strengths and limitations

This paper is based on our reflections of conducting a large cluster RCT in care home settings. Other study teams may have encountered different challenges, and we also encourage similar reflective pieces to improve care home research. Some of our experiences and the legal frameworks and infrastructure within which we worked are specific to England, although our experiences may provide some lessons for researchers in other, similar, settings. This was a large and inclusive study in care homes and the study team made considerable efforts to be inclusive both of care homes and residents in the study (e.g. simplifying language and processes, providing tailored succinct training). We were only able to introduce use of nominated consultees partway through the study and no breakdown is available on how many consultees were personal versus nominated, which would be useful to include in future research to better inform RECs.

## 4 Conclusion

We have outlined some of the challenges in relation to consent that we experienced whilst conducting the AFRI-c study and aiming to be as inclusive as possible in recruitment. We would also like to emphasise that support from national infrastructure (i.e. the ENRICH/RDN teams) was often essential to care homes’ successful participation. Despite the high-pressure environment and the additional time burden, there were many positive and encouraging displays of commitment to research from both care home staff and residents. Our experience suggests that care homes are largely willing and engaged with the idea of research. Conducting research in care homes is a rewarding experience, however there are considerable gaps in the current research infrastructure for care homes, making it challenging to conduct research in the setting.

We hope that by sharing our experiences, researchers in the future may benefit from our findings. Care homes are an important setting to include in research and it is crucial that we find solutions to the challenges, rather than using them to justify the exclusion of care homes as a research setting. It is also essential that we enable all residents in care homes to have the opportunity to participate in research to improve the generalisability, and benefit, of research.

## Funding

This trial was funded by the National Institute for Health and Social Care (NIHR) Public Health Research (PHR) Programme NIHR129783. The views expressed are those of the author(s) and not necessarily those of the NIHR or the Department of Health and Social Care. Versuni (previously Philips) provided the air filtration units at a discounted rate. Neither the funder nor Versuni (previously Philips) have any involvement in the design of the trial, data collection, analysis or interpretation of the data, decision to publish or preparation of the manuscript.

## Declaration of competing interest

The authors declare the following financial interests/personal relationships which may be considered as potential competing interests:

Alastair Hay reports financial support was provided by National Institute for Health and Care Research for this research. The other authors declare that they have no known competing financial interests or personal relationships that could have appeared to influence the work reported in this paper.
